# Pedicle Dysplasia in Proximal Thoracic Adolescent Idiopathic Scoliosis Curves: What are We Missing and What are its Possible Surgical Implications? An Observational Retrospective Study on 104 Patients

**DOI:** 10.1177/21925682241230964

**Published:** 2024-02-06

**Authors:** Giovanni Viroli, Alberto Ruffilli, Francesca Barile, Marco Manzetti, Matteo Traversari, Cesare Faldini

**Affiliations:** 1 Department of Biomedical and Neuromotor Science - DIBINEM, University of Bologna, 1st Orthopaedic and Traumatologic Clinic, 18509 IRCCS Istituto Ortopedico Rizzoli, Bologna, Italy

**Keywords:** pedicle dysplasia, adolescent idiopathic scoliosis, lenke classification, proximal curve, shoulder balance, shoulder imbalance

## Abstract

**Study Design:**

Retrospective cohort study.

**Objectives:**

To assess if pedicle dysplasia is present in proximal thoracic (PT), both structural and nonstructural, compared to main thoracic (MT) curves; and to assess if it is predictive of radiographic outcomes at minimum 2 years of follow-up.

**Methods:**

A retrospective review of surgically-treated Adolescent Idiopathic Scoliosis (AIS) patients with Lenke 1-2-3-4 curves was performed. On preoperative CT-scan, at the apical vertebra, pedicle width on the concavity (PWc) and on the convexity (PWv) and Pedicle Dysplasia Index (PDI, defined as PWc/PWv) were measured. Preoperative and last follow-up (at least 2 years) x-rays were reviewed.

**Results:**

104 patients meeting the inclusion criteria were divided into Structural-PT (S-PT) and Nonstructural-PT (NS-PT) groups based on Lenke criteria. PWc (*P* < .001). And PDI (*P* < .001 for S-PT, *P* = .004 for NS-PT) were significantly smaller in the PT than in MT curves for both groups. PT-PWc significantly correlated with follow-up PT Cobb for both groups (*P* < .001 and *P* = .015 respectively). PT-PDI significantly correlated with follow-up PT-Cobb (*P* < .001), CA (*P* < .040) and T1 tilt (*P* < .002), only for NS-PT group. NS-PT patients with PWc PT <1 mm had higher RSHD (*P* = .021) and T1 tilt (*P* = .025) at follow-up. NS-PT patients with PDI PT <.3 had higher RSHD (*P* < .001), CA (*P* = .002) and T1 tilt (*P* = .003) at follow-up.

**Conclusion:**

S-PT and NS-PT curves show significant pedicle dysplasia on the concavity. Pedicle dysplasia significantly correlated with shoulder balance at follow-up, for NS-PT patterns. Patients with a PWc <1 mm or PDI <.30 are at particular risk of postoperative shoulder imbalance.

## Introduction

Adolescent idiopathic scoliosis (AIS) is a complex tridimensional spinal deformity characterized by typical anatomopathological features, such as translation, rotation and wedge-shaped deformation of the vertebral bodies. Furthermore, pedicles on the concave side of the curves are characterized by a narrower diameter compared to pedicles on the convex side.^1^ While these features are keystones elements in the definition of the AIS curves and in the concept of curve structuration (as they are more marked toward the apex of the curve and as curve severity increases), none of the most widely adopted classification systems.^2,3^ takes them into account. In particular, pedicle dysplasia has gained growing attention with the development and spreading of pedicle screws systems, since it could be determinant in the choice of screws’ diameter and trajectory.^4^ Its prevalence, although based only upon the absolute pedicle width, is reported to be significantly higher in AIS patients than in the general population.^5^ However, while pedicle dysplasia has been extensively described in main thoracic curves (MT),^6^ it has not been thoroughly characterized in proximal thoracic curves (PT). Therefore, the first aim of the presented study is to analyze the presence of pedicle dysplasia in PT curves, both structural and nonstructural according to the Lenke classification’s criteria.

In the second place, the study looked at any possible connection between pedicle dysplasia and the main surgical outcomes, particularly with regard to PT curve correction and shoulder balance, at minimum 2 years of follow-up.

## Patients and Methods

### Study Design

A retrospective review of AIS patients with a major thoracic curve pattern (Lenke 1, 2, 3, 4) who underwent correction surgery between 2016 and 2020 was undertaken. Non-idiopathic scoliosis, infantile and juvenile idiopathic scoliosis diagnoses, or age>25, were considered exclusion criteria, as well as the absence of a preoperative CT-scan. Follow-up of less than 2 years was considered an exclusion criterion. All patients signed an informed consent on the use of their clinical documentation for scientific purposes. The local Ethics Committee approved this retrospective study.

### Data Collection

**Figure 1.**
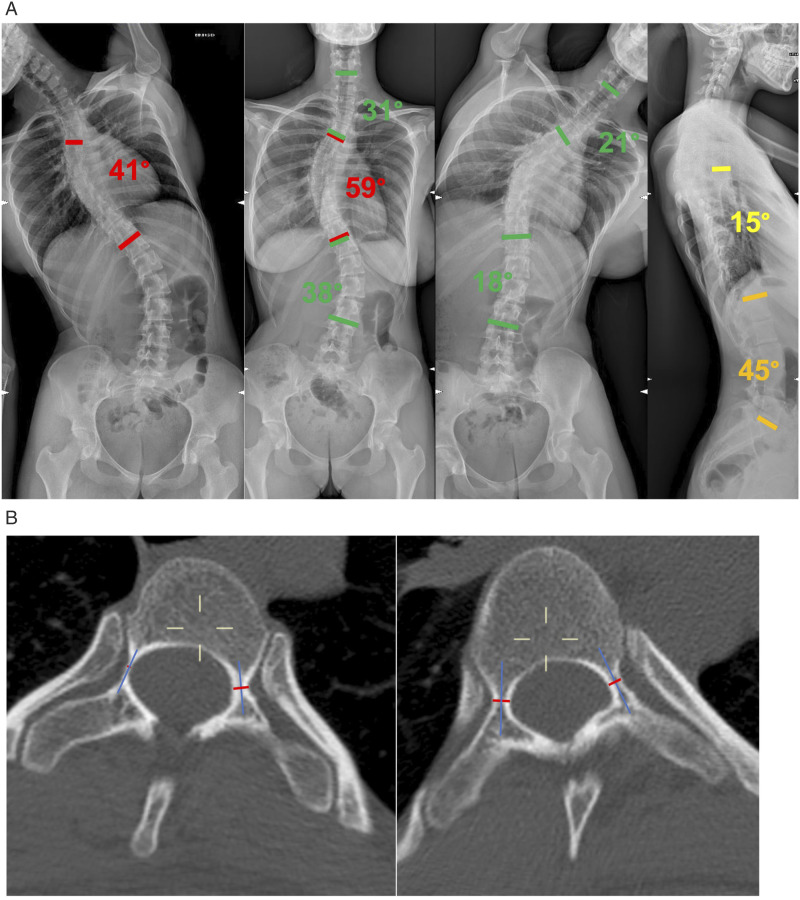
Exemplificative case. (A): Preoperative radiographic appearance of a 20 years old female patient with a 59° main thoracic curve. The side bending and lateral films, reveal the unstructural nature of the proximal thoracic and of the lumbar curves (B): Axial CT-scan of apical vertebra of the PT curve (T1, left) and of the MT curve (T7, right). In blue, the pedicle axis, in red the PW (pedicle width). It can be noted the severe pedicle dysplasia in the PT curve (PWc = 0,4 mm; PWv = 2,8 mm) and the moderate dysplasia in the MT curve (PWc = 2,4; PWv = 2,9 mm).

The pedicle anatomy was studied using preoperative multiplanar reformation (MPR) CT scan images. Window level and diameter were optimized for the measurement of bony structures. Using the multiplanar reformation, we chose the optimal slice trying to obtain the most precise image in the true axial plane of the apical vertebra, at the level of the middle portion of the pedicle in the craniocaudal direction. Pedicle width on the concavity (PWc) and on the convexity (PWv) side at the apical vertebra of the PT and MT curves were measured, defined as the shortest distance along the perpendicular to the line passing through the pedicle’s axis, using the outer cortex of the pedicles as reference (Figure 1). Moreover, Pedicle Dysplasia Index (PDI), defined as PWc/PWv, was introduced as a new parameter in order to further assess the anatomical dysplasia of pedicles. Finally, on preoperative MPR CT scan, the angle of rotation (RAsag) of the apical vertrebra was measured. On the preoperative and last follow-up full-length standing x-rays, the following measures were taken: Cobb angle of PT and MT curve, T5-T12 kyphosis (TK), L1-S1 lordosis (LL), radiographic shoulder height difference (RSHD), clavicle angle (CA), T1 tilt, C7 plumb line/central sacral vertical line distance (C7PL-CSVL) and MT curve apical vertebral translation (AVT). All the measurements were taken by two experienced spine surgeons. All patients underwent scoliosis correction based on the same technique: posterior approach, high density pedicle screws, multiple asymmetrical periapical Ponte osteotomies in the MT curve, translation maneuver over asymmetric shaped cobalt chrome rods and direct vertebral rotation. The implants used to treat these patients were K2M Mesa ® and Solera Medtronic ®. All surgeries were performed by the same surgical team. The fusion area was selected according to Lenke’s criteria.^2^

### Patients Characteristics

104 patients (85 females and 19 males) were included, with a follow-up of 38.8 ± 15.1 (range 24-72). The average age was 15.6 ± 3.1 (range 11-24). Patients were subdivided in Structural PT group (S-PT), which included patients with a structural PT curve according to Lenke criteria (2 and 4 pattern), and Nonstructural PT group (NS-PT), which included patients with a nonstructural PT curve according to Lenke criteria (1 and 3 pattern). Patient’s characteristics are summarized in Table 1. None of the included patients had a history of significant shoulder injuries, diseases or of shoulder surgeries.

**Table 1.** Sample Characteristics, Including Clinical and Radiographic Features.

Patients n	104 (85 Females; 19 Males)
Average age (years)	15.63 ± 3.14 (range 11-24)
Curve type	I: 38; II: 31; III: 27; IV 8
Average follow-up (months)	38.80 ± 15.14 (range 24-72)
Preoperative PT cobb	35.23 ± 16.00
Preoperative MT cobb	69.29 ± 23.04
Preoperative TK	23.54 ± 17.29
Preoperative LL	55.87 ± 13.95
Postoperative PT cobb	22.09 ± 10.44
Postoperative MT cobb	26.45 ± 11.67
Postoperative TK	21.35 ± 10.68
Postoperative LL	52.29 ± 10.16

### Statistical Analysis

Parametric test was used to compare samples in case of continuous variables, normal distribution and appropriate numerousness. As parametric test, two-tailed student*t* test was used to compare the mean values of the variables. Multivariate regression analysis was utilized to identify the possible correlations between pedicle dysplasia and radiographical outcomes. *P* values <.05 were considered to be significant. Jamovi statistical analysis software (The jamovi project (2021), jamovi Version 1.6) was used to perform statistical analysis.

## Results

### Pedicle Dysplasia and Apical Vertebral Rotation of Structural and Nonstrutcutal Proximal Thoracic Curves

PWc in the PT curve resulted significantly lower than PWc in the MT curve both in all cohort (1.63 ± .86 vs 3.27 ± 1.33; *P* < .001), S-PT group (1.42 ± .71 vs 3.44 ± 1.46; *P* < .001) and NS-PT group (1.76 ± .93 vs 3.18 ± 1.24; *P* < .001). Conversely, PWv did not significantly differ between PT and MT regions, in all the three groups. PDI proved to be significantly smaller in the PT curves than in MT curves, both in all cohort (.39 ± .22 vs .80 ± .95; *P* < .001), S-PT (.36 ± .27 vs .72 ± .29; *P* < .001) and NS-PT curves (.41 ± .18 vs .85 ± 1.18; *P* = .004). Conversely, RASag proved to be significantly higher in the MT curves than in PT curves, both in all cohort (10.06 ± 5.4 vs 20.22 ± 9.93; *P* < .001) S-PT group (10.06 ± 5.4 vs 20.22 ± 9.93; *P* < .001) and NS-PT group (10.06 ± 5.4 vs 20.22 ± 9.93; *P* < .001). Finally, the S-PT group and the NS-PT group were compared for each of the aforementioned parameters. Of note, PWc PT was found to be significantly smaller in the S-PT group (1.42 ± .71 vs 1.76 ± .93; *P* = .036). Moreover, both RASag PT and RASag MT were significantly higher in the S-PT group than in the NS-PT group (13.05 ± 5.50 vs 8.33 ± 4.60, *P* < .001; 25.36 ± 10.64 vs 17.31 ± 8.26 *P* < .001) (Table 2).

**Table 2.** Pedicle Dysplasia and Apical Vertebral Rotation in PT and MT Curves.

	n	PWc PT	PWc MT	*P* value	PWv PT	PWv MT	*P* value	PDI PT	PDI MT	*P* value	RASag PT	RASag MT	*P* value
All Cohort	104	1.63 ± .86	3.27 ± 1.33	< .001***	4.29 ± .96	4.83 ± 3.59	.139	.39 ± .22	.80 ± .95	< .001***	10.06 ± 5.43	20.22 ± 9.93	< .001***
S-PT group	65	1.42 ± .71	3.44 ± 1.46	< .001***	4.40 ± 1.15	4.76 ± 1.10	.163	.36 ± .27	.72 ± .29	< .001***	13.05 ± 5.50	25.36 ± 10.64	< .001***
NS-PT group	39	1.76 ± .93	3.18 ± 1.24	< .001***	4.22 ± .83	4.88 ± 4.48	.252	.41 ± .18	.85 ± 1.18	.004**	8.33 ± 4.60	17.31 ± 8.26	< .001***
P value (S-PT vs NS-PT groups)		.036*	.357		.413	.837		.243	.407		< .001***	< .001***	

*P < .05, **P < .01, ***P < .001. PWc (pedicle width on the concavity), PWv (pedicle width on the convexity), PDI (pedicle dyslasia index: PWc/PWv), RASag (angle of rotation), PT (proximal thoracic curve), MT (main thoracic curve), S-PT group (structural proximal thoracic group), NS-PT group (nonstructural proximal thoracic group). The bold indicates stastically significant values.

### Is Pedicle Dysplasia in the Pt Curve A Potential Predictor for Correction Results?

**Image 1.**
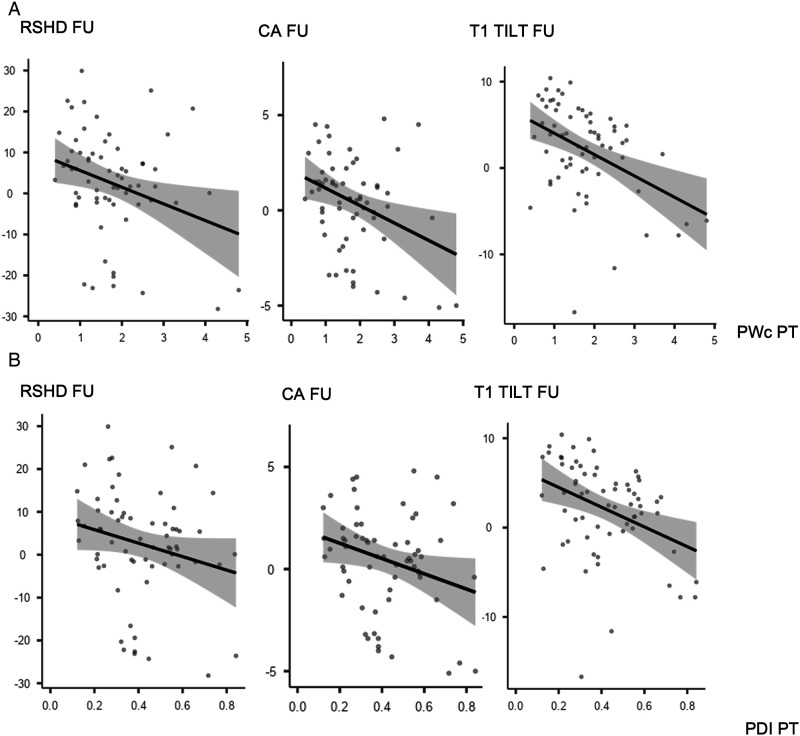
(A and B) Correlation plots between PWc (2A), PDI (2B) and shoulder balance parameters (Radiographic Shoulder Height Difference, Clavicular Angle, T1 Tilt) for Nonstructural PT group.

**Image 2.**
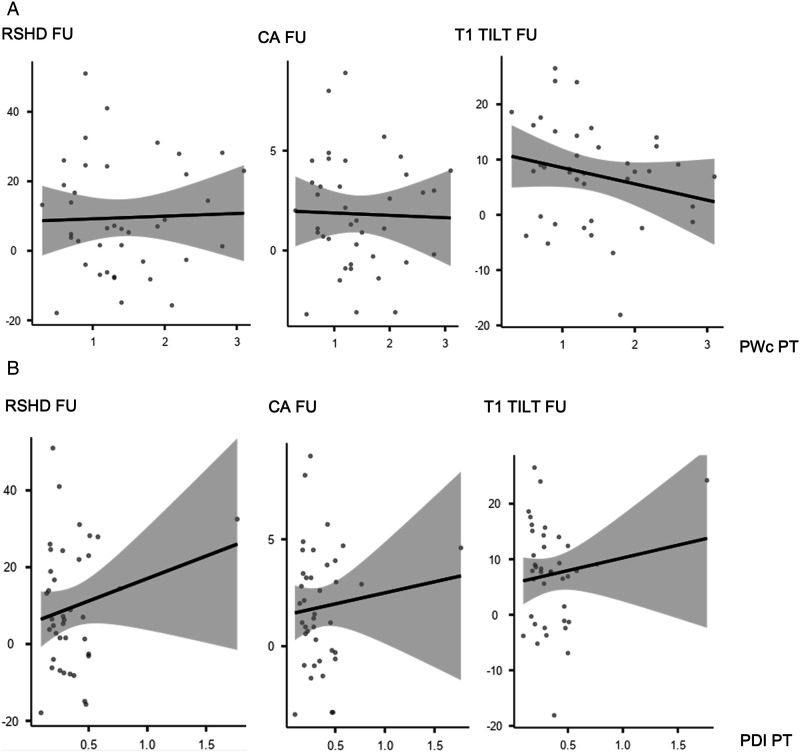
(A and B) Correlation plots between PWc (3A), PDI (3B) and shoulder balance parameters (Radiographic Shoulder Height Difference, Clavicular Angle, T1 Tilt) for Structural PT group.

PWc in the PT curve was significantly correlated with Last Follow-up Cobb of the PT curve both in all cohort (R = −.384, *P* < .001), S-PT group (R = −.520, *P* < .001) and NS-PT group (R = −.302, *P* < .015). Moreover, PWc of the PT curve was significantly correlated with all the shoulder balance measures in the last follow-up x-rays for all cohort (RSHD: R = −.319, *P* < .048; CA: R = −.215, *P* < .017; T1 tilt: R = −.335, *P* < .001) and NS-PT group (RSHD: R = −.296, *P* < .017; CA: R = −.322, *P* < .009; T1 tilt: R = −.420, *P* < .001) (Image 1(A)), but not for S-PT group (Image 2(A)). PDI of the PT curve showed significant correlation with Last FU radiographic outcomes only in NS-PT (Image 1(B) and 2(B)) group, and specifically for the following parameters: Last Follow-up Cobb of the PT (R = −.425, *P* < .001), CA (R = −.255, *P* < .040) and T1 tilt (R = −.372, *P* < .002) (Table 3). To further assess the relation between dysplasia and shoulder balance, patients in S- and NS-PT groups were stratified based on PWc (< or ≥1 mm) and PDI (< or ≥.3) (Table 4). NS-PT group patients with PWc PT <1 mm had significantly higher RSHD (6.11 ± 7.88 vs 3.27 ± 1.33; *P* = .021) and T1 tilt (3.50 ± 4.48 3.18 ± 1.24; *P* = .025) at 2 years follow-up. NS-PT patients with PDI PT <.3 had significantly higher RSHD (2.57 ± 9.38 vs −.53 ± 12.79; *P* < .001), CA (−.54 ± 1.66 vs −.07 ± 2.78; *P* = .002) and T1 tilt (2.78 ± 4.24 vs .98 ± 5.34; *P* = .003) at 2 years follow-up.

**Table 3.** Correlation Between PWc, PDI, RASag and Radiographic Parameters for PT Curve.

		RASag PT pre-op		RASag MT pre-op		Cobb PT FU		Cobb MT FU		TK FU		LL FU		C7PL-CSVL FU		RSHD FU		CA FU		T1 Tilt FU		AVT FU	
		Pearson’s R	*P*-value	Pearson’s R	*P*-value	Pearson’s R	*P*-value	Pearson’s R	*P*-value	Pearson’s R	*P*-value	Pearson’s R	*P*-value	Pearson’s R	*P*-value	Pearson’s R	*P*-value	Pearson’s R	*P*-value	Pearson’s R	*P*-value	Pearson’s R	*P*-value
PWc PT	All Cohort **cohort**	−.373	< .001***	−.027	.775	−.384	< .001***	−.153	.092	−.117	.199	−.156	.087	−.011	.908	−.179	.048*	−.215	.017*	−.345	< .001***	−.163	.074
	S-PT **-PT**	−.159	.347	.041	.817	−.520	< .001***	−.197	.230	−.057	.732	.021	.898	−.201	.219	.034	.838	−.030	.858	−.223	.173	−.308	.057
	NS-PT	−.359	.004**	.077	.554	−.302	.015*	−.054	.667	−.076	.548	−.241	.055	.056	.656	−.296	.017*	−.322	.009**	−.429	< .001***	.023	.859
PWv PT	All cohort	−.052	.578	.204	.030*	−.121	.185	.045	.626	.066	.472	−.105	.253	.034	.713	−.136	.137	−.088	.336	−.138	.130	.023	.799
	S-PT	−.182	.280	.099	.571	−.337	.036*	−.135	.414	.058	.724	−.016	.925	.037	.821	−.111	.501	−.062	.706	−.183	.265	.149	.364
	NS-PT	.047	.711	.264	.038*	.078	.538	.164	.191	−.038	.763	−.171	.177	−.091	.469	−.224	.073	−.214	.087	−.189	.132	.123	.329
PDI PT	All cohort	−.215	.020*	−.094	.324	−.144	.113	−.102	.264	−.105	.251	−.126	.170	.019	.832	−.030	.745	−.106	.244	−.119	.194	−.138	.128
	S-PT **-PT**	.190	.259	−.075	.667	.178	.278	.043	.796	−.041	.804	−.123	.457	−.036	.827	.199	.224	.101	.541	.135	.414	−.236	.149
	NS-PT	−.457	< .001***	−.003	.979	−.425	< .001***	−.155	.219	−.045	.723	−.179	.158	.108	.393	−.222	.076	−.255	.040*	−.372	.002**	−.015	.905
RASag PT pre-op	All cohort	—	—	.073	.440	.556	< .001***	.451	< .001***	.315	< .001***	−.015	.869	−.102	.270	.147	.112	.170	.066	.264	.004**	.220	.017*
	S-PT **-PT**	—	—	−.222	.201	.306	.065	.394	.016*	.339	.040*	−.109	.521	.053	.754	.082	.630	.085	.615	.038	.825	−.032	.853
	NS-PT	—	—	.112	.385	.522	< .001***	.336	.007**	.281	.024*	−.081	.526	−.282	.024*	.076	.549	.083	.514	.274	.028*	.174	.169

*P < .05, **P < .01, ***P < .001. PWc (pedicle width on the concavity), PWv (pedicle width on the convexity), PDI (pedicle dyslasia index: PWc/PWv), RASag (angle of rotation), TK (T5-12 thoraci kyphosis), LL (L1-S1 lumbar lordosis), C7PL-CSVL (C7 plumb line/central sacral vertical line distance), RSHD (radiographic shoulder height difference), CA (clavicle angle), AVT (apical vertebral translation), PT (proximal thoracic curve), MT (main thoracic curve), S-PT group (structural proximal thoracic group), NS-PT group (nonstructural proximal thoracic group). The bold indicates stastically significant values.

**Table 4.** Stratification of S- and NS-PT Groups Based on PWc (< or ≥1 mm) and PDI (< or ≥.3).

	Nonstructural PT	Nonstructural PT	*P* value	Nonstructural PT	Nonstructural PT	*P* value	Structural PT	Structural PT	*P*value	Structural PT	Structural PT	*P*value
	PWc PT <1 mm	PWc PT ≥ 1 mm		PDI PT <0.3	PDI PT ≥ 0.3		PWc PT <1 mm	PWc PT ≥ 1 mm		PDI PT <0.3	PDI PT ≥ 0.3	
*RSHD*	6.11 ± 7.88	3.27 ± 1.33	.021*	2.57 ± 9.38	−.53 ± 12.79	<.001***	13.56 ± 15.85	7.10 ± 15.20	.236	9.79 ± 15.15	8.56 ± 16.42	.813
*CA*	1.24 ± 1.55	3.44 ± 1.46	.054	−.54 ± 1.66	−.07 ± 2.78	**0.002	2.57 ± 2.74	1.45 ± 2.83	.245	2.18 ± 2.87	1.37 ± 2.76	.378
*T1 Tilt*	3.50 ± 4.48	3.18 ± 1.24	.025*	2.78 ± 4.24	.98 ± 5.34	**0.003	10.21± 10.57	5.84 ± 8.48	.210	9.59 ± 8.68	4.33 ± 9.54	.085

*P < .05, **P < .01, ***P < .001. The bold indicates stastically significant values.

Furthermore, for all the three groups, RASag of the PT curve significantly correlated with several radiographic parameters at last follow-up. Table 3. When looking at MT curves, some significant correlations were found for PWc MT with Cobb Angle of the MT curve and TK at the last follow-up of the NS-PT group, and for PDI MT with Last Follow-up LL for all cohort and NS-PT group. Table 5.

**Table 5.** Correlation Between PWc, PDI, RASag and Radiographic Parameters for MT Curve.

		RASag PT Pre-op		RASag MT Pre-op		Cobb PT FU		Cobb MT FU		TK FU		LL FU		C7PL-CSVL FU		RSHD FU		CA FU		T1 Tilt FU		AVT FU	
		Pearson’s R	*P*-value	Pearson’s R	*P*-value	Pearson’s R	*P*-value	Pearson’s R	*P*-value	Pearson’s R	*P*-value	Pearson’s R	*P*-value	Pearson’s R	*P*-value	Pearson’s R	*P*-value	Pearson’s R	*P*-value	Pearson’s R	*P*-value	Pearson’s R	*P*-value
PWc MT	All cohort	−.044	.637	.156	.098	.043	.639	−.116	.205	−.099	.280	.035	.701	.101	.267	.053	.560	.059	.519	.072	.431	.037	.688
	Structured PT	−.135	.427	.227	.190	.248	.129	−.151	.360	.036	.826	.116	.482	.072	.661	.147	.372	.147	.373	.045	.785	.129	.434
	Non structured PT	−.171	.176	.052	.688	−.184	.143	−.255	.040*	−.382	.002**	−.018	.890	.201	.108	.097	.443	.085	.502	.101	.422	.073	.563
PWv MT	All cohort	−.007	.938	.184	.051	.015	.873	.008	.933	−.023	.802	−.133	.145	.124	.174	−.062	.494	−.053	.562	−.050	.587	.047	.608
	Structured PT	.043	.801	.195	.263	.200	.221	.171	.297	−.049	.769	−.221	.177	.114	.490	−.057	.732	−.103	.531	−.311	.054	−.108	.514
	Non structured PT	−.014	.910	.282	.026*	.028	.822	.006	.965	−.016	.898	−.193	.127	.192	.125	−.045	.722	−.030	.815	.014	.911	.125	.321
PDI MT	All cohort	−.093	.317	−.040	.671	−.017	.849	−.112	.218	−.145	.110	−.197	.030*	.021	.821	.043	.641	.030	.742	.048	.597	.019	.837
	Structured PT	−.201	.234	.133	.445	.224	.171	−.259	.111	.011	.949	.196	.233	−.005	.975	.239	.143	.275	.091	.284	.080	.158	.336
	Non structured PT	−.099	.437	−.059	.650	−.024	.849	−.107	.396	−.227	.069	−.347	.005**	.054	.670	.059	.638	.038	.764	.056	.657	.027	.829
RASag MT pre-op	All cohort	.073	.440	-	-	.236	.012*	.402	< .001***	.161	.089	.119	.211	.114	.229	−.001	.991	−.036	.703	−.091	.340	.147	.121
	Structured PT	−.222	.201	-	-	.055	.752	.151	.388	−.029	.868	.397	.018*	.290	.091	−.120	.492	−.187	.281	−.286	.096	.370	.029*
	Non structured PT	.112	.385	-	-	.151	.242	.505	< .001***	.170	.187	−.197	.128	−.119	.356	−.098	.449	−.100	.441	−.341	.007**	.070	.587

*P < .05, **P < .01, ***P < .001. PWc (pedicle width on the concavity), PWv (pedicle width on the convexity), PDI (pedicle dyslasia index: PWc/PWv), RASag (angle of rotation), TK (T5-12 thoraci kyphosis), LL (L1-S1 lumbar lordosis), C7PL-CSVL (C7 plumb line/central sacral vertical line distance), RSHD (radiographic shoulder height difference), CA (clavicle angle), AVT (apical vertebral translation), PT (proximal thoracic curve), MT (main thoracic curve), S-PT group (structural proximal thoracic group), NS-PT group (nonstructural proximal thoracic group. The bold indicates stastically significant values.

## Discussion

The gold standard of AIS surgical classifications is the Lenke classification system.^2^ The funding concept of this classification system is the distinction between structural and nonstructural curves, which is based on the magnitude of the curves measured on side bending films or on the presence of kyphosis at the involved tract. Structural curves remain greater than 25° on side bending or they are characterized by segmental kyphosis greater than 20° (at T2-T5 for PT curves, at T10-L2 for MT or thoracolumbar/lumbar curves).

As a general principle, but with some exceptions, structural curves need to be fused while nonstructural curves may not be included in the fusion area. In fact, unfused nonstructural curves typically show spontaneous correction with the correction of the adjacent structural curve.^7,8^ However, the presence of the typical anatomopathological features of structural curves in a minor nonstructural curve, could raise questions about its real nature, whether structural or nonstructural, regardless of its side bending flexibility. Consequently, this may influence the treatment in terms of fusion area or corrective maneuver. For example, in some Lenke 1C curves, it may be advised to perform a non-selective thoraco-lumbar fusion if there is a substantial AVR in the nonstrucural lumbar curve (AVR ratio MT: TL/L ≃ 1).^9^ Alongside rotation, a key and often overlooked pathological element of AIS vertebrae is pedicle dysplasia. While pedicle dysplasia has been extensively described in MT curves, its presence in structural and nonstructural PT curves is still unclear. In particular, Çatan et al.^10^ found a smaller pedicle width in the concavity of the MT curve compared to the convex side of AIS curves, but the same result was not found at the PT level. Conversely, Takeshita et al,^11^ in a subset of scoliotic patients of mixed aetiology, and Kuraishi et al ,^12^ in a group of AIS patients, demonstrated that pedicles on the concave side of PT curve was significantly dysplastic, with smaller diameters compared to the convex side. The first aim of our study was to bring further evidence regarding the possible presence of typical AIS pathoanatomical features, particularly in terms of pedicle dysplasia and AVR, in the PT curves, both structural and nonstructural. In the present study, the analysis of a single-center-109 AIS patients cohort, confirmed the presence of AVR and pedicle dysplasia in the PT curves, both structural and nonstrucural, introducing a new parameter, the PDI. In the second place, and to our knowledge for the first time, a significant correlation between pedicle dysplasia and radiographic correction outcomes, particularly in terms of shoulder balance and PT curve coronal correction, has been showed at 2 years follow-up.

### Limitations

The present study does not come without several limitations. Firstly, the measures were manually performed using multiplanar reformation (MPR) CT scan images. Considering the tridimensional nature of the scoliotic deformity, obtaining a perfectly oriented image in the axial plane of the vertebrae may at times be challenging and minor variations can lead to major measurement differences. Secondly, all the patients included in the present study had a preoperative CT-scan for surgical planning reasons. Considering the risks of radiation exposure, this examination is not routinely performed for all patients at our center. Typically, the decision to perform a preoperative low-dose CT-scan for surgical planning is made on a case-by-case basis, depending on the patient’s age and on the curve’s characteristics (magnitude, rotation, stiffness). Therefore, our cohort may only represent a limited subset of surgical AIS patients. However, while this may be a bias as for the first question (more severe curves may have more severe pathoanatomical features, in terms of dysplasia and AVR), we believe that the correlation between pedicle dysplasia and radiographic outcomes is especially relevant when planning a challenging AIS correction case. Thirdly, the single-center-, single-surgical team-design is a clear limitation: some key point like the surgical technique adopted must be acknowledged as possible confounding factors. Moreover, we only considered radiographic shoulder balance, without a clinical shoulder balance evaluation, which may be inconsistent with radiographic shoulder balance.^13^ In addition to it, we were not able to perform a correlation between the radiographic results and the outcome of a well refined questionnaire about the cosmetic perception of each patient, specifically focusing on the individual impact of shoulder balance. In fact, no questionnaire such as the Spinal Appearance Questionnaire.^14^ was validated in our language at the time of the enrollment. Finally, the retrospective nature of the study is a clear limitation. Despite that, compared to previous studies on this topic, possible strengths of our study are the relatively large sample, the inclusion of Lenke 1-4 patterns, of male and female patients, and of all-AIS curves.

### Pedicle Dysplasia and Apical Vertebral Rotation of Structural And Nonstrutcutal Proximal Thoracic Curves

The present study demonstrated a clear presence of the typical scoliotic anatomopathological features at the level of the minor, proximal curve. Regarding the pedicle dysplasia, PT curves’ concavity, in both S- and NS-PT groups, showed a significantly narrower diameter in the axial plane (PWc) than MT curves’ concavity. However it must be considered that PW is reported to decrease from T1 to T4 and to increase from T5 to T12.^5^ Therefore, to reduce any size variation associated with the multiple levels being evaluated, we introduced a new parameter named pedicle dysplasia index (PDI), which is defined as PWc/PWv. The analysis of the PDI of the PT and MT curves, confirmed the existence of a more severe dysplasia at the level of the PT curve apex, both structural and nonstructural. Regarding the presence of significant dysplasia at the PT level, our results agree with those of Takeshita et and Kuraishi et al, but with an increased level of evidence, thanks to a more homogeneous cohort (only AIS curves) and a larger sample size (104 patients). Moreover, our results partially contrast with those of Gao et al,^5^ who, in a cohort of Lenke 1 patients, found the structural MT curve to be more severely dysplastic than the nonstructural PT curve. In fact, we found the PT curve, even when nonstructural, to be more severely dysplastic than the MT curve, in terms of reduced PWc. This is somewhat unexpected and paradoxical. In fact, we typically imagine the PT as a curve that develops as a mere mechanical compensation to the MT curve, allowing the patients to maintain a horizontal gaze. Mechanics undoubtedly has an impact on pathoanatomy since, according to the Hueter-Volkmann principle, the forces that arise on a compensatory curve might lead to a reduced growth on the concavity-compression, side, compared to the convexity-tension, side.^15^ However, the fact that pedicle dysplasia in a NS-PT curve is even worse than in a structural MT curve, suggests the possibility that this might be the result of a biologic pathoanatomical process that is primitively active in the upper thoracic vertebrae and not a simple consequence of the mechanical environment in which the vertebrae are developing.

On the other hand, rotation was shown to be significantly worse in MT than PT curves, in both S and NS-PT groups. These findings seem to suggest that, from a pathoanatomical perspective, the MT curve might be “rotation driven”, whereas the PT curve might be “dysplastic driven”.

Finally, S- and NS-PT curves were compared. The S-PT curves had more severe pathological anatomy features than NS-PT curves when it came to rotation (*P* < .001) and dysplasia, though there was a minor statistical significance for PWc (*P* = .036) and no significance for PDI (*P* = .243). This confirmed that the severity of the pathoanatomical features is proportional to the structuring nature of the deformity.

### Is Pedicle Dysplasia in the Pt Curve A Potential Predictor for Correction Results?

To our knowledge for the first time, the present study searched for a possible correlation between the pedicle dysplasia, particularly in the PT curve, and radiographic correction outcomes at minimum 2 years of follow-up. In particular, a significant correlation between the PWc in the PT curve and the magnitude of the PT curve at the last follow-up occurred, for both S- and NS-PT curve. The fact that this correlation was stronger for S-PT than for NS-PT group (R = −.520, *P* < .001 vs R = −.302, *P* < .015) may suggest that it may have been biased by the curve’s stiffness. Nevertheless, the fact that both the S-PT (relatively stiffer) and NS-PT (relatively more elastic) groups achieved this outcome indicates that additional variables are at work.

In particular, this may have a mechanical explanation, since in case of severely dysplastic pedicles, funnel pedicle screw trajectories are hardly feasible, and in-out-in trajectories are often required.^16,17^ However, in-out-in screws inevitably provide lower pullout resistance, ranging from 64 to 80%.^18–20^ of the interpedicular screws’ pullout strength. This is clearly a limitation in terms of the amount of corrective force that can be demanded to each anchor point, either objective (suboptimal force transmission from the screw to the vertebra) or subjective (lower exertion of corrective forces by the surgeon, trying to avoid any intraoperative pull-out).

Finally, one of the most interesting findings of this study, was the identification of a correlation between PT curve pedicle dysplasia and radiographic shoulder balance at minimum 2 years of follow-up. Shoulder balance has been identified as a key aspect of idiopathic scoliosis aesthetic deformity.^21,22^ In particular Raso et al.^23^ reported that shoulder imbalance, combined with scapular and waist asymmetry, accounted for 75% of the perception of trunk deformity by the patients. At the same time, restoring an ideal shoulder balance after AIS surgery can be challenging, as the reported postoperative shoulder imbalance rate is nearly 25% .^24^ Moreover, the identification of preoperative risk factors for postoperative shoulder imbalance is still an unsolved question, as a recent meta-analysis identified the Lumbar Curve preoperative Cobb to be the only significant risk factor.^24^ In our study, at minimum 2 of years follow-up, shoulder balance was significantly correlated with pedicle dysplasia only for NS-PT group. Specifically, both medial and lateral shoulder balance were affected, with RSHD significantly related with PWc (R = −.296, *P* < .017), CA significantly related with PWc (R = −.322, *P* < .009) and PDI (R = −.255, *P* < .040), T1 tilt significantly related with PWc (R = −.420, *P* < .001) and PDI (T1 tilt: R = −.372, *P* < .002). Conversely, no significant correlation between any radiographic shoulder balance parameter and pedicle dysplasia was recorded for S-PT group. This data are even more interesting when considering that pedicle dysplasia was significantly more severe in S-PT group than NS-PT group, as previously stated. These results might be explained by a classification issue. In other words, in Lenke 2 and 4 patterns, structural PT curves are usually appropriately addressed thanks to a fusion area usually extended up to T2 and/or via an effective corrective maneuver (more accurate segmental compression-distraction at the upper thoracic level). This would eventually lead to a more balanced correction rate between the MT and PT curves and a reduction of the T1 tilt, with a consequent improvement in shoulders balance.

Conversely, in Lenke 1 and 3 patterns, as PT curves are reported to self-correct in 86% of patients,^25^ nonstructural PT curves are hardly included in the fusion area and the corrective maneuver is more focused on the MT curve, but this may rise some shoulder balance issues. In fact, not only a correlation between pedicle dysplasia and shoulder balance was detected, but the present study identified a specific subset of Lenke 1 and 3 patterns, characterized by a severe pedicle dysplasia in the PT curve (PWc < 1 mm or PDI <.3), which are at particular risk of shoulder imbalance at 2 years FU. Lenke classification still provides an invaluable guide in choosing fusion levels, but in this specific subset of patients, during corrective maneuver, extreme care should be taken to achieve a balanced correction between PT and MT curve, as long as to achieving a good reduction of T1 tilt. In the event that these two goals cannot be achieved with a UIV at T3 or lower, fusion to T2 should be considered. Postoperative shoulder imbalance is a highly-complex and likely multifactorial phenomenon, but pedicle dysplasia could be another aspect to be considered in a multifactorial model in order to reduce as much as possible the risk of shoulder imbalance. Future, prospective, research is needed in order to confirm our results and to prove their clinical significance.

## Conclusion

Proximal thoracic curves, both structural and nonstructural, show significant pedicle dysplasia on the concave side, which resulted to be more severe than what is observed in the main thoracic curves. Secondly, a significant correlation was found between the pedicle width on the PT curves’ concavity and the shoulder balance at 2 years follow-up, for nonstructural PT patterns. In particular, patients with a PWc <1 mm and PDI <.30 are at particular risk of postoperative shoulder imbalance. Our results must be interpreted in light of the study limitations and future studies with a more refined design should further confirm the results of this preliminary study. Furthermore, we hope that this may be the prelude to a new classification system for AIS, which ideally should be three-dimensional and should consider the pathological anatomy of the curves.
